# Supplementary material for: Nuclear Morphological Remodeling in Human Granulocytes Is Linked to Prenylation Independently from Cytoskeleton
Source: Cells. 2020 Nov 20;9(11):2509. doi: 10.3390/cells9112509 (PMC7699803; doi:10.3390/cells9112509)
Supplement: Supplementary file 1 [file cells-09-02509-s001.zip › cells-980065-supplementary.publication/SUPPLEMENTARY FILE.pdf]

Nuclear morphological remodeling in human granulocytes is linked to prenylation independently from cytoskeleton

Authors: *Sebastian Martewicz[1], Camilla Luni[1], Xi Zhu[1,2,3], Meihua Cui[1], Manli Hu[1], Siqi Qu[1,2], Damiano Buratto[1], Guang Yang[1], Eleonora Grespan[4], Nicola Elvassore[1,5,6,7]**

[1] Shanghai Institute for Advanced Immunochemical Studies (SIAIS), ShanghaiTech University, 393 Middle Huaxia Road, Pudong New District, Shanghai 201210, China

[2] School of Life Science and Technology, ShanghaiTech University, 393 Huaxia Road, Shanghai, 201210, China

[3] University of Chinese Academy of Sciences, 19A Yuquan Road, Beijing, 100049, China

[4] Institute of Neuroscience, National Research Council, Padova, 35127, Italy

[5] Department of Industrial Engineering, University of Padova, via Marzolo 9, 35131 Padova, Italy

[6] Venetian Institute of Molecular Medicine, via Orus 2, 35129 Padova, Italy

[7] Stem Cells & Regenerative Medicine Section, UCL Great Ormond Street Institute of Child Health, 30 Guilford Street, London WC1N 1EH, UK

*****Corresponding author address:

E-mail: nicola.elvassore@unipd.it
Shanghai Institute for Advanced Immunochemical Studies
ShanghaiTech University
393 Huaxia Road, Shanghai 201210, China
Telephone: +39 049 827 5469
Fax: +39 049 827 7599

SUPPLEMENTARY INFORMATION

Contents:

Page 2 – Movie S1-S4 captions
Page 2 – Description of Supplementary Excel data files "Datasets"
Pages 3-5 – Extended Materials and Methods section with References
Pages 6-25 – Supplementary Figures S1-S20
Pages 26-27 – Supplementary Tables

Movie S1 – Z-stacks of ER and nuclei staining in Day 3 iHL60 treated with 10 µM AY9944. 3D reconstruction of representative nuclei with aberrant lobulated morphology. Asterisks correlated z-stack and 3D reconstructed nuclei.

Movie S2 – Z-stacks of ER and nuclei staining in Day 3 iHL60 treated with 5 µM GGTI-298. 3D reconstruction of representative nuclei with aberrant lobulated morphology. Asterisks correlated z-stack and 3D reconstructed nuclei.

Movie S3 – Z-stacks of ER and nuclei staining in Day 3 iHL60 treated with 20 µM FTI-277. 3D reconstruction of representative nuclei with aberrant lobulated morphology. Asterisks correlated z-stack and 3D reconstructed nuclei. Orange arrows point at nuclear segment connections, represented by thin filaments in control cells and large nuclear constrictions in treated cells.

Movie S4 – Z-stacks of ER and nuclei staining in Day 3 iHL60 treated with 5 µM L-778,123. 3D reconstruction of representative nuclei with aberrant lobulated morphology. Asterisks correlated z-stack and 3D reconstructed nuclei.

Dataset 1 – Gene list and clustering of DEG in the "Microtubule cytoskeleton" Cellular Component category. Color Coding maintained as in Figure S8A. Results of enrichment analyses for each cluster group according to Biological Process categories.

Dataset 2 – Gene list and clustering of DEG in the "Actin cytoskeleton" Cellular Component category. Color Coding maintained as in Figure S8B. Results of enrichment analyses for each cluster group according to Biological Process categories.

Dataset 3 – Gene list and clustering of DEG in the "Nuclear Envelope" Cellular Component category. Color Coding maintained as in Figure 3A. Results of enrichment analyses for each cluster group according to Biological Process categories.

Dataset 4 – Complete data for expression levels of RNA transcripts in HL60 and iHL60 cells.

Dataset 5 – Results of enrichment analyses of DEGs between the 3 comparisons (Day 2 vs. Day 0; Day 4 vs. Day 0; Day 2 vs. Day 4) according to Biological Process, Cellular Component, Molecular Function and Reactome categories.

Dataset 6 – Results of enrichment analyses of DEGs clustered according to expression temporal profile according to Reactome categories. Cluster numbering is conserved with Figure 3C.

Extended Materials and Methods

Cell Cultures. HL60 cells were obtained from ECACC (Sigma-Aldrich, cat#98070106) and maintained at 37°C in a humidified 5% CO_2 environment in RPMI 1640 (ThermoScientific) supplemented with 10% FBS (ThermoScientific). Cultures were passaged every 2 days at 10^5 cells/ml density. Stocks of cells frozen in 90% FBS + 10% DMSO (Sigma-Aldrich) were thawed in complete medium and tested negative for mycoplasma contamination (MycoAlert™ PLUS Mycoplasma Detection Kit, Lonza). Induction of granulocytic differentiation was performed by addition of 5 µM all-*trans*-retinoic acid (ATRA) (Sigma-Aldrich) at Day 0 to $2x10^5$ cell/ml cultures. For RNA sample collection, at Day 2 iHL60 cultures were diluted 1:5 with fresh medium, as this step halves apoptotic cells at Day 4 without affecting final cell yield, providing more robust results. Biological replicates refer to samples derived from independent differentiation protocols of subsequent culture passages between p7-p20, accounting for 1 month of continuous culture.

RNA extraction, sequencing and PCR. Total RNA was isolated at 0, 48 and 96 hours of ATRA treatment from 10^7 cells with TRIzol Reagent (ThermoScientific) followed by purification with RNeasy Mini Kit (Qiagen), and quantifying by NanoDrop (ThermoScientific). 5 µg of sample were further processed at GeneWiz, Suzhou, China. The library products were ready for sequencing via Illumina HiSeq 2x150PE, with 10G coverage, producing ~40.7M clean reads per sample on average. For single transcript level analyses, total RNA was retrotranscribed with High Capacity cDNA Reverse Transcription kit (ThermoFisher Scientific) and real-time PCR was performed with PowerUp™ SYBR™ Green Master Mix (ThermoScientific) for 40 cycles in a QuantStudio 6 Flex thermocycler (ThermoScientific). Primer sequences are reported in Table S1.

Bioinformatics analyses. Reads were mapped against the human genome GRCh38.89 by using RSEM 1.3.0 with STAR 2.6.1a (Li and Dewey, 2011). Only genes with gene IDs in the current NCBI annotation were retained. The identified genes were filtered out if not expressed at a count-per-million (CPM) higher than 1 in at least 2 samples of at least one time-point. The final number of genes used in subsequent analyses is 12308 genes. Data were normalized using the trimmed mean of M-values normalization method (TMM) implemented in edgeR Bioconductor package (version 3.22.4) (Robinson and Oshlack, 2010). Differentially expressed genes (DEGs) were computed with edgeR, using a mixed criterion based on p-value, after false discovery rate (FDR) correction by Benjamini-Hochberg method, lower than 0.05 and absolute log2(fold change) higher than 2. A Principal Component Analysis was performed by Singular Value Decomposition (SVD) on log2(CPM+1) data, after centering, using MATLAB R2017a (The MathWorks). DEGs enrichment analysis of Gene Ontology (GO) categories was performed using clusterProfiler Bioconductor package (version 3.10.1) (Yu et al., 2012), imposing an FDR-corrected p-value < 0.01. DEGs enrichment analysis of Reactome pathways (updated in July 2019) was performed using ClueGO (version 2.5.4) (Bindea et al., 2009) and CluePedia (version 1.5.4) (Bindea et al., 2013). A MATLAB script was written to cluster the temporal profiles of DEGs according to their log2-fold change between subsequent time points, imposing an absolute cutoff value of 0.4 to determine a significant change in expression. Hierarchical clustering was performed with Euclidean distance and complete linkage using median-centered data, and plotted as heat maps in MATLAB.

All data are publicly available in GEO repository, accession number GSE134922.

Drug treatments. For each drug employed, targets and references are reported in Table S2. Cells were exposed for 3 hours, 24 hours or 3 days (as indicated in the text) at various drug concentrations (as indicated in the text). *Cytoskeletal drugs.* Latrunculin A (Sigma-Aldrich), Cytochalasin D (Cayman), Jasplakinolide (Cayman), Nocodazole (Sigma-Aldrich), Vincristine sulfate (Selleck), Paclitaxel (Selleck), Monomeric Acrylamide (Sigma-Aldrich), β-β'iminodipropionitrile (Sigma-Aldrich), Withaferin A (Sigma-Aldrich), Y-27632 (Selleck), Blebbistatin (Selleck), 2,4,6-Triiodophenol (Bidepharm), Pentabromopseudilin (Sigma-Aldrich), Pentachloropseudilin (Aobious).

Cholesterol biosynthesis pathway. Lovastatin (Selleck), Simvastatin (Selleck), Zoledronate (Selleck), Alendronate (Selleck), Ketoconazole (Selleck), AY9944 (Selleck), U18666A (Selleck), Triparanol (Selleck), FTI-277 (Selleck), GGTI-298 (Selleck), 17α-hydroxyprogesterone (APExBIO), L-778,123 (Targetmol). In double treatment experiments, all compounds were administered simultaneously, with the exception of 3-day long experiments, in which cells were pre-treated for 1 hour with either latrunculin A or Y-27632 before vincristine sulfate supplementation.

Cell live imaging. Cell nuclei were visualized by incubation for 15 minutes at 37°C with 1 µg/ml Hoechst 33342 (Cell Signaling) in culture medium. The endoplasmic reticulum was stained by 30-minute incubation at 37°C with 2 mM ER-Tracker™ Blue-White DPX (ThermoScientific) in culture medium. Cells were centrifuged for 3 minutes at 300x*g* to increase cell density, deposited on a glass-bottom multiwell plate, clamped with a glass coverslip and imaged within 30 minutes with an inverted Zeiss LSM 710 laser-scanning confocal microscope, 100x oil-immersion objective, 405 nm excitation wavelength and a 0.5 µm step.

Image analyses. For volume and surface quantification, images of nuclei stained with ER-Tracker™ were processed with the Image Processing Toolbox of MATLAB software (R2015b). Each analysis involved z-stacks of approximately 30 steps and at least 30 cells per day per replicate (6 biological replicates). Image processing for quantification included contrasting, black and white transformation and exclusion of cells located at image borders. Cell identity between consecutive z-stacks was tracked by calculating the centroid coordinates of each virtual object within an image and assigning same identity to centroids with less than 30% variation relative to the diameter of the virtual object of the central z-stack section. Aberrantly assigned objects and dead or mitotic cells were manually excluded from analyses. *Quantification nuclear volume and surface:* a negative black and white image of ER-Tracker™ staining was employed. Perimeter and area of each object calculated using MATLAB functions *bwlabel* and *regionprops*. Segmented lobules identified as separate objects were considered belonging to one nucleus when their centroid distance was less than 50% the cell diameter calculated at the central stack. Voxel volume was 0.083x0.083x0.5 µm = 0,0034445 μm^3. *Quantification of cell volume:* the formula for a spherical volume $\boldsymbol{V} = \frac{4}{3}\pi r^3$ was employed with $r = \frac{dmax}{2}$, where *dmax* is the longest diameter for any section of an individual cell.

Qualitative evaluation of nuclear lobulation. The "Number of lobules" was manually derived for each nucleus from Hoechst33342 staining images and plotted as count distribution for number of lobules. The "Maximum number of sections" was manually derived from ER-Tracker staining images by considering the maximum number of nuclear sections in a cell for any given focal plane in the z-stack, and plotted as count distribution for number of sections.

For qualitative analyses, the three categories were defined as "Round/Ovoid", according to geometry, "Segmented" if the nucleus presented at least 2 well defined separated volumes, and "Deformed" when neither of the previous two applied. Qualitative evaluation is presented as percentage on total population for each class.

Flow cytometry analyses. 5×10^5 cells were collected for each staining, centrifuged for 3 minutes at 300x*g* and washed twice with ice-cold PBS+10% FBS. Live immunostaining was performed in 100 µl for 40 minutes on ice in the dark with the antibody concentration suggested by the supplier and washed twice with ice-cold PBS+10% FBS. All antibodies were from Biolegend: CD11b (cat#301309), CD54 (cat#353105), CD62L (cat#304803). Respiratory burst assay was performed according to manufacturer's instructions (Cayman, cat#601130). Samples were analyzed with the CytoFLEX Platform (Beckman Coulter) analyzing at least 10^4 single cell events.

Molecular modeling. The models for the FTase, GGTase I, GGTase II and GGTase III were derived from published crystal structures [Protein Data Bank (PDB) ID codes 1S63, 1S64, 3DSS and 6J74/6J7X respectively]. The missing residues of each structure have been modeled using the MODELLER package (Sali and Blundell, 1993). The model of the complex GGTaseII with two PO_4^{3-} ions was made

starting from the crystal structure of the complex GGTase II-GGPP [PDB ID: 3DST]. The two ions were superposed to the pyrophosphate atoms of the GGPP and the GGPP was then removed. The atomic coordinates for the 3D structure of the L-778,123 were obtained starting from the smile code, with hydrogen atoms addition and structure minimization done using ChemOffice18 packaged. Docking has been performed using AutoDock4ZN package (Morris et al., 2009; Santos-Martins et al., 2014).

Statistical analyses. Data for cell and nuclear areas and volumes are presented as median and interquartile range (IQR). Lognormal data distribution was normalized and statistical analyses were performed by one-way ANOVA followed by Tukey–Kramer test in computing environment R. Data for lobule and section numbers are presented as lognormal distributions of counts per cell normalized for width. Statistics were performed by non-parametric Kruskal-Wallis test with post-hoc Dunn test for pairs. Data Plotting and statistics were performed with Origin 2020 software.

Bindea, G., Galon, J., and Mlecnik, B. (2013). CluePedia Cytoscape plugin: pathway insights using integrated experimental and in silico data. Bioinformatics (Oxford, England) *29*, 661-663.

Bindea, G., Mlecnik, B., Hackl, H., Charoentong, P., Tosolini, M., Kirilovsky, A., Fridman, W.H., Pages, F., Trajanoski, Z., and Galon, J. (2009). ClueGO: a Cytoscape plug-in to decipher functionally grouped gene ontology and pathway annotation networks. Bioinformatics (Oxford, England) *25*, 1091-1093.

Li, B., and Dewey, C.N. (2011). RSEM: accurate transcript quantification from RNA-Seq data with or without a reference genome. BMC bioinformatics *12*, 323.

Morris, G.M., Huey, R., Lindstrom, W., Sanner, M.F., Belew, R.K., Goodsell, D.S., and Olson, A.J. (2009). AutoDock4 and AutoDockTools4: Automated docking with selective receptor flexibility. J Comput Chem *30*, 2785-2791.

Robinson, M.D., and Oshlack, A. (2010). A scaling normalization method for differential expression analysis of RNA-seq data. Genome biology *11*, R25.

Sali, A., and Blundell, T.L. (1993). Comparative protein modelling by satisfaction of spatial restraints. J Mol Biol *234*, 779-815.

Santos-Martins, D., Forli, S., Ramos, M.J., and Olson, A.J. (2014). AutoDock4(Zn): an improved AutoDock force field for small-molecule docking to zinc metalloproteins. J Chem Inf Model *54*, 2371-2379.

Yu, G., Wang, L.G., Han, Y., and He, Q.Y. (2012). clusterProfiler: an R package for comparing biological themes among gene clusters. Omics : a journal of integrative biology *16*, 284-287.

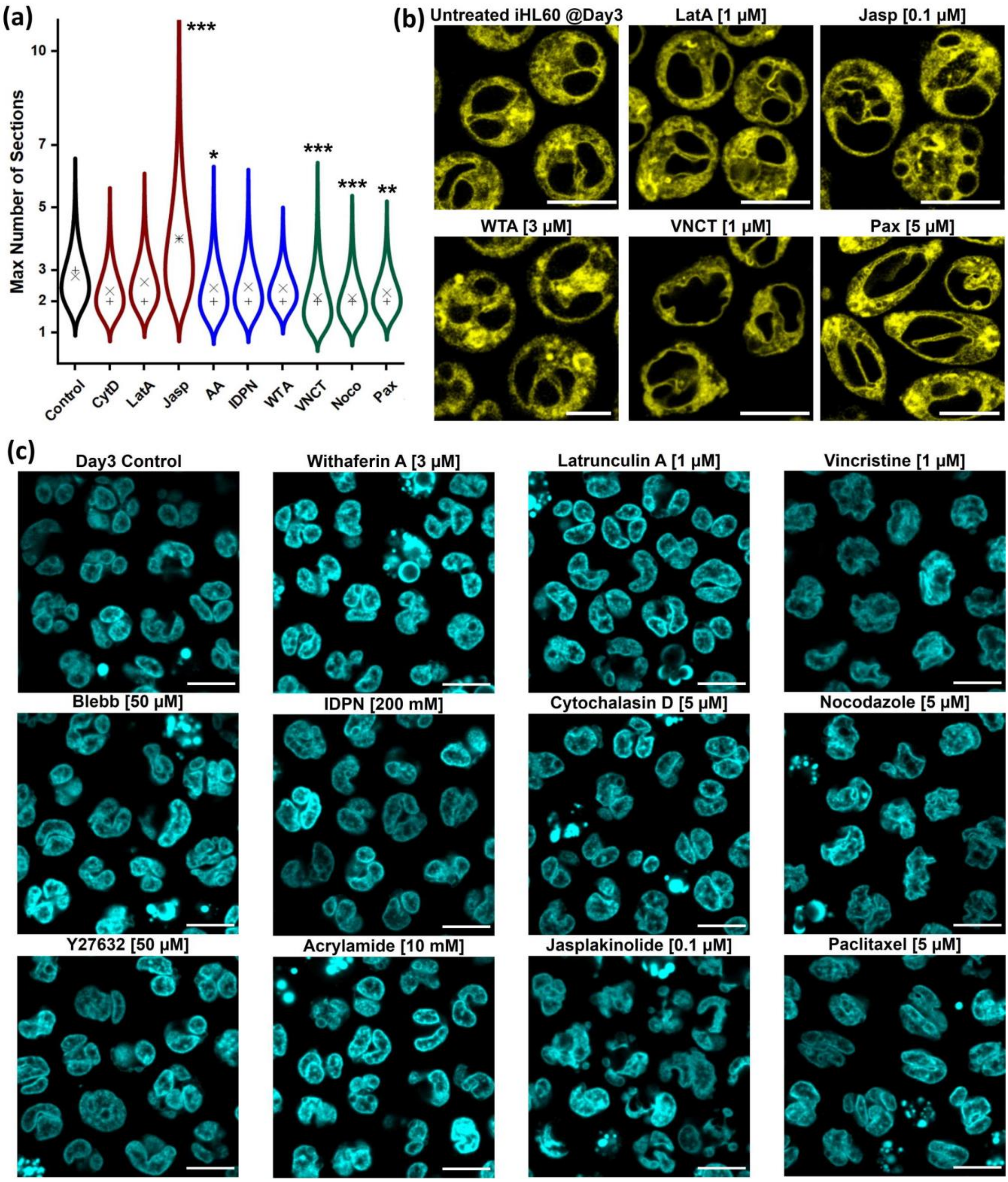

Figure S1 (a) Quantification of maximum number of nuclear sections per nucleus in iHL60 at Day3 according to treatment (a minimum of 78 cells for condition were quantified) (× = mean; + = median; *** = p<.001, Kruskal-Wallis test with Dunn post-hoc comparison). **(b)** Live ER staining of Day 3 iHL60 (scale bar = 10 µm), treated for 3 hours with the specified cytoskeleton modifying drugs. **(c)** Live DNA staining of Day 3 iHL60 (scale bar = 10 µm), treated for 3 hours with the specified cytoskeleton modifying drugs.

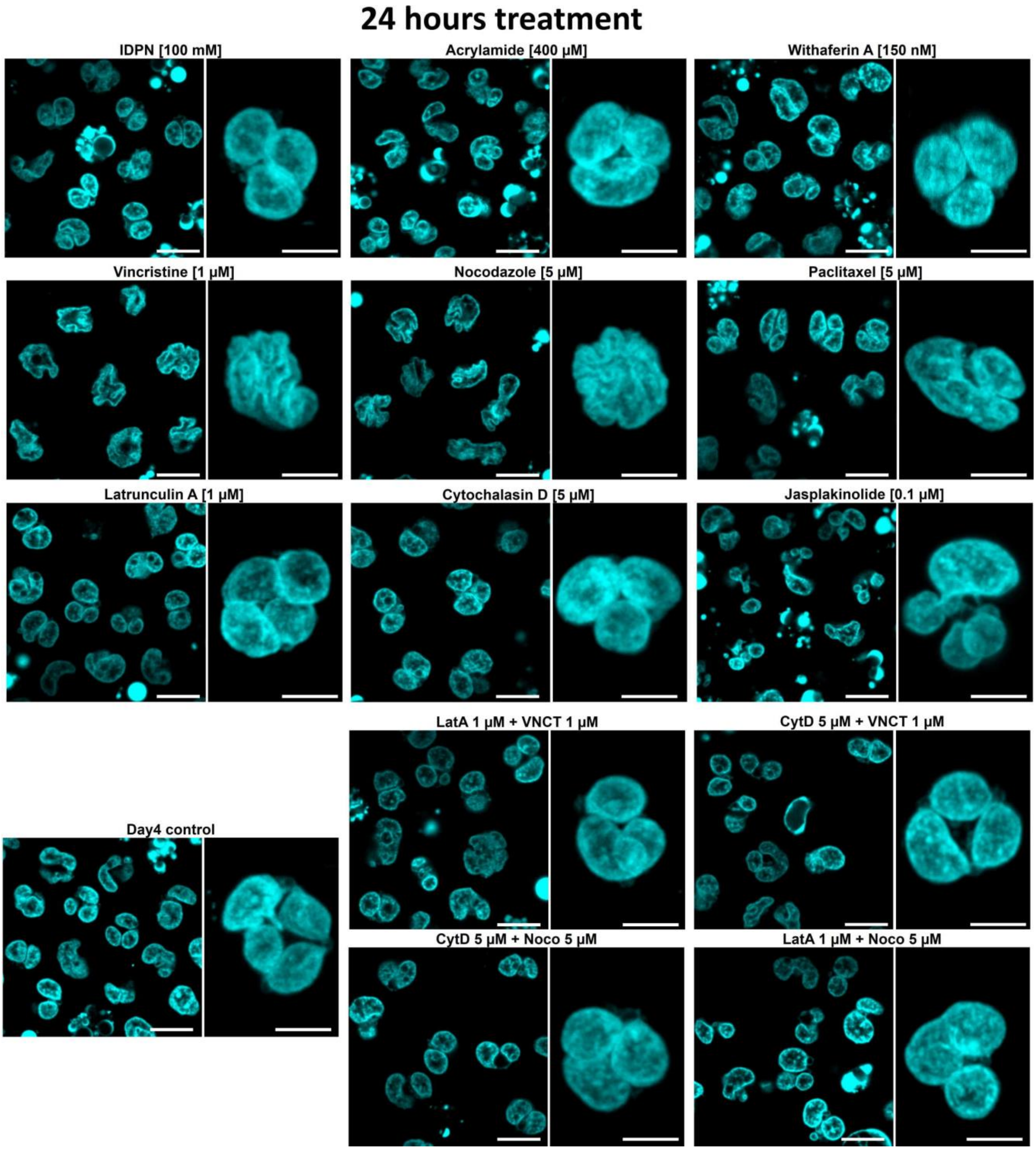

Figure S2 Live DNA staining of Day 4 iHL60 (scale bar = 10 μm, inset scale bar = 5 μm), treated for 24 hours with the specified cytoskeleton modifying drugs.

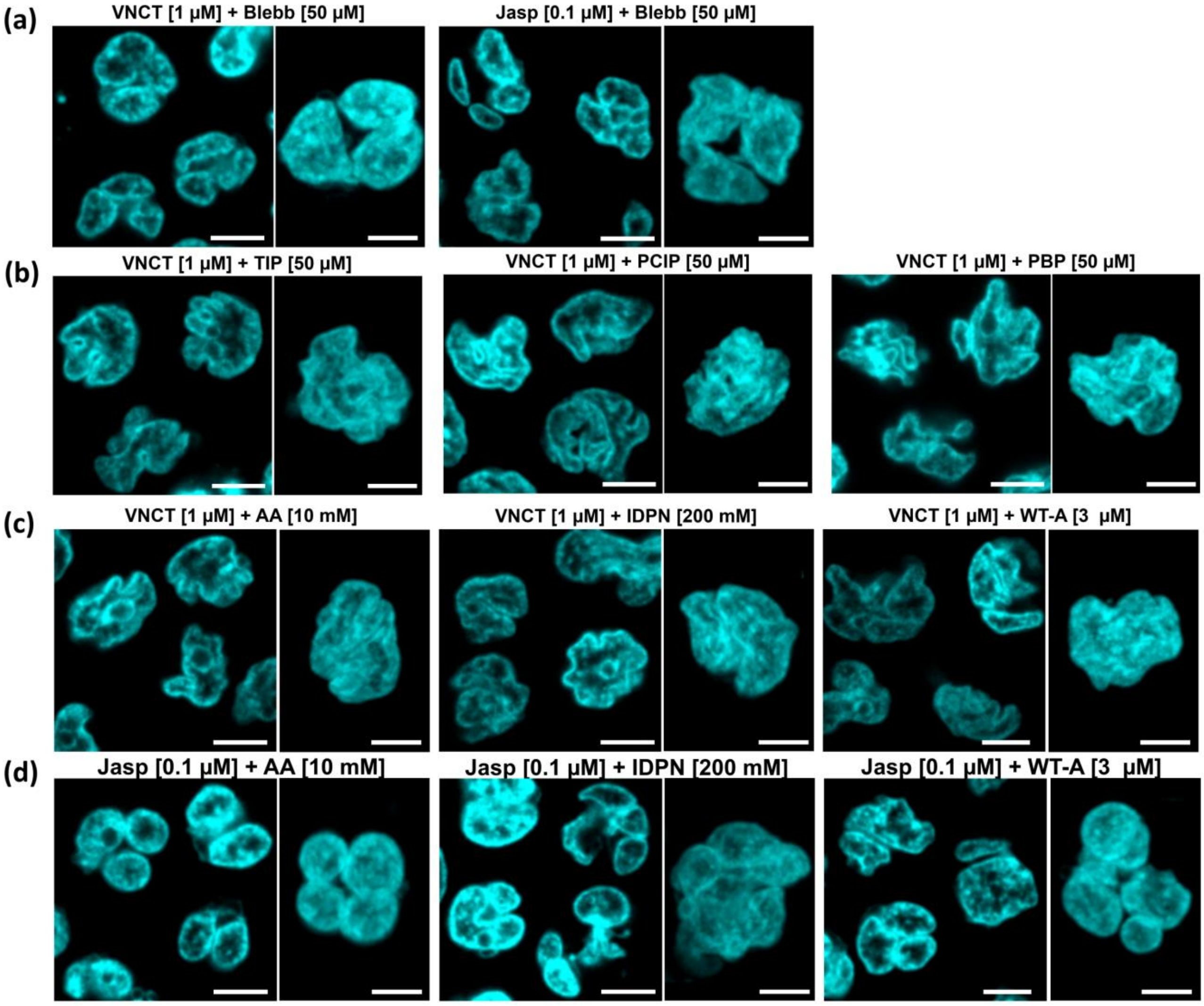

Figure S3 (a-d) Live DNA staining of Day 3 iHL60 (scale bar = 5 µm), treated for 3 hours with the specified cytoskeleton modifying drugs and drug-combinations.

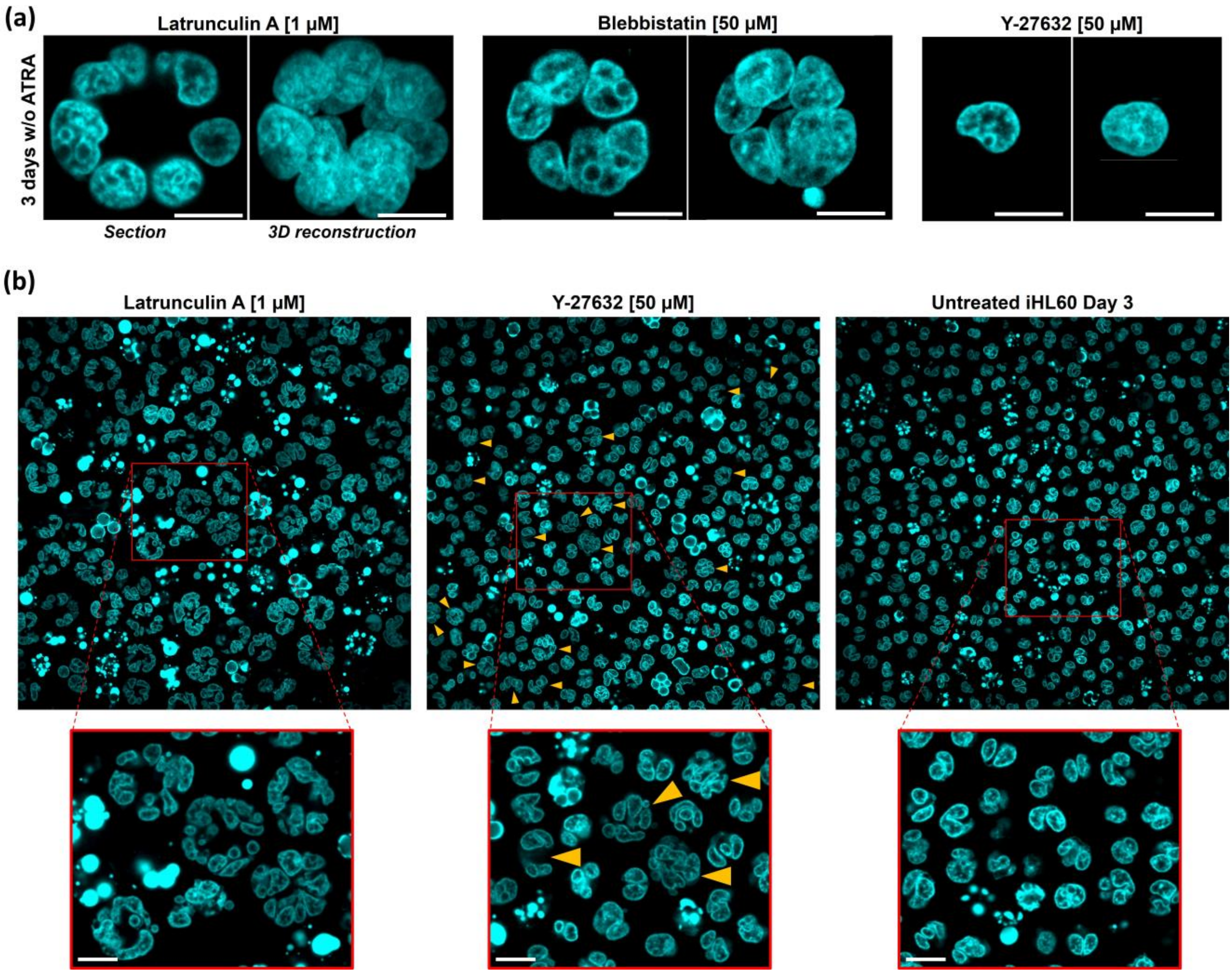

Figure S4 (a-b) Live DNA staining of Day 3 iHL60, treated for 3 days with the specified cytoskeleton modifying drugs (scale bars = 10 µm). Orange arrowheads in **(b)** indicating medium-sized cells after partial cytokinesis failure.

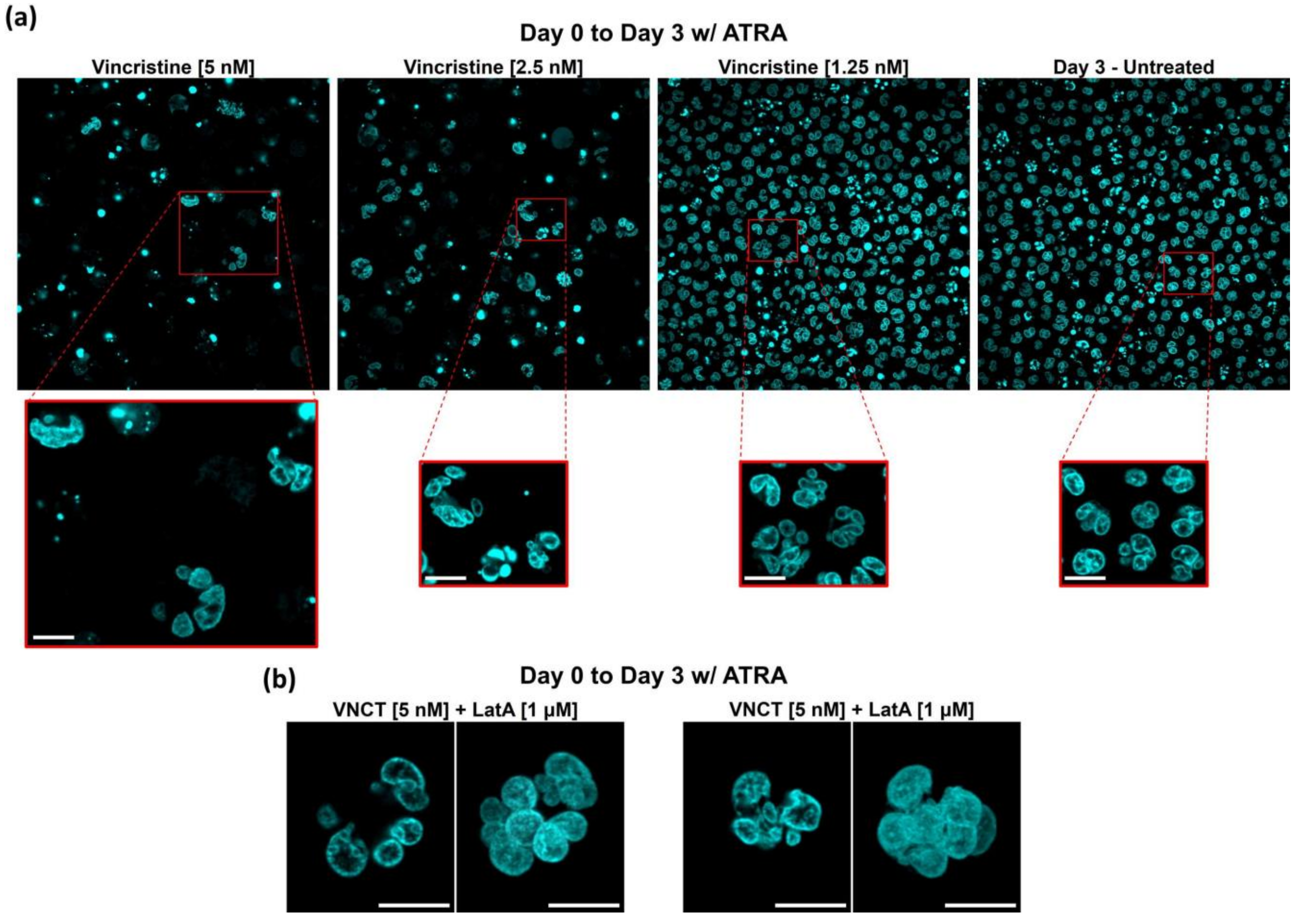

Figure S5 Live DNA staining of Day 3 iHL60, treated for 3 days with the specified cytoskeleton modifying drugs (scale bars = 10 μm).

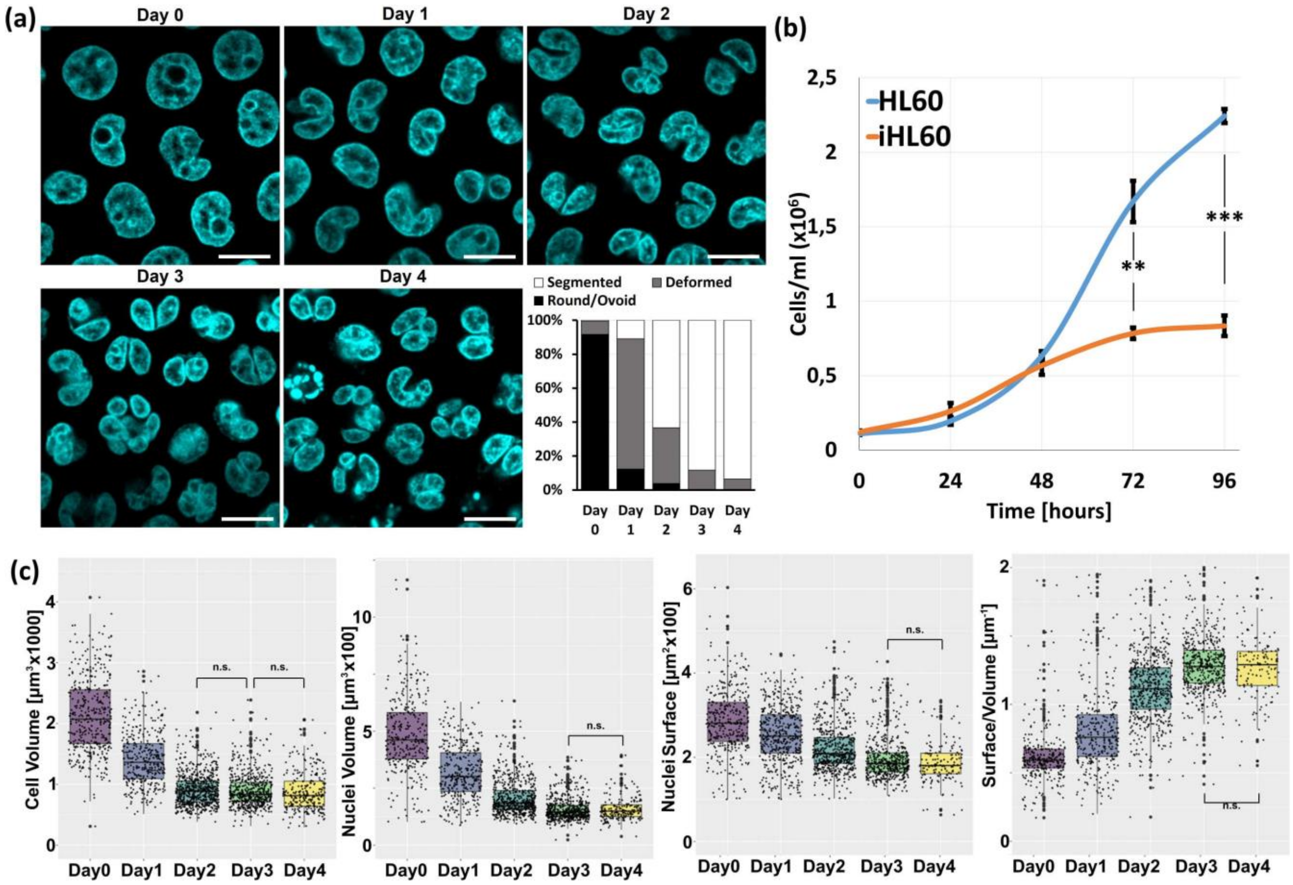

Figure S6 (a) Live DNA staining of HL60 at different time-point of differentiation, with qualitative nuclear morphology assessment (scale bar = 10 µm). (b) Proliferation curve of HL60 and iHL60 (n = 3 independent cultures). (c) Quantification of cellular and nuclear dimensions at different time-points of iHL60 differentiation (n = 6 biological replicates, at least 30 cells *per* day *per* replicate).

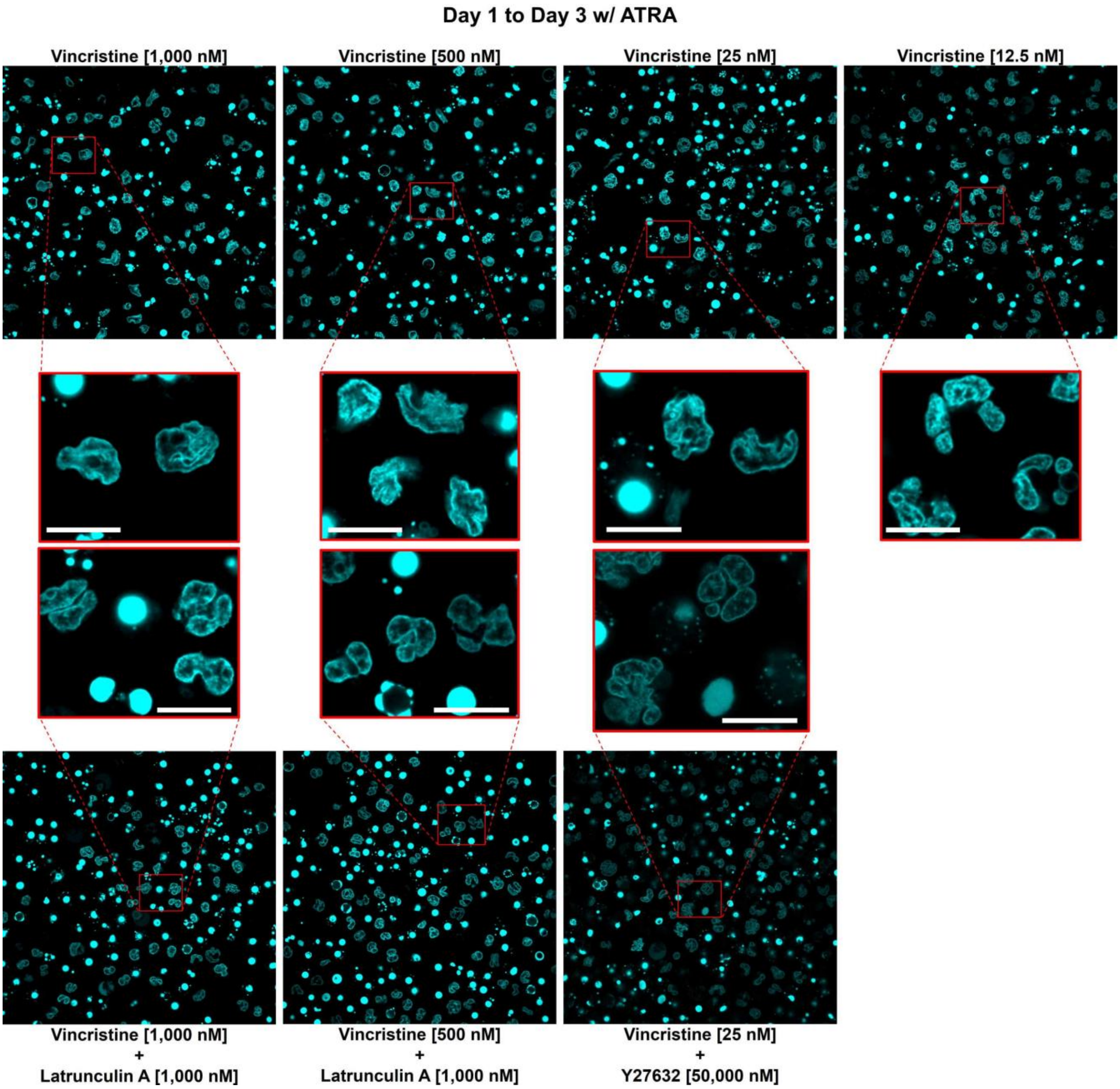

Figure S7 Live DNA staining of Day 3 iHL60, treated for 2 days with the specified cytoskeleton modifying drugs (scale bars = 10 µm).

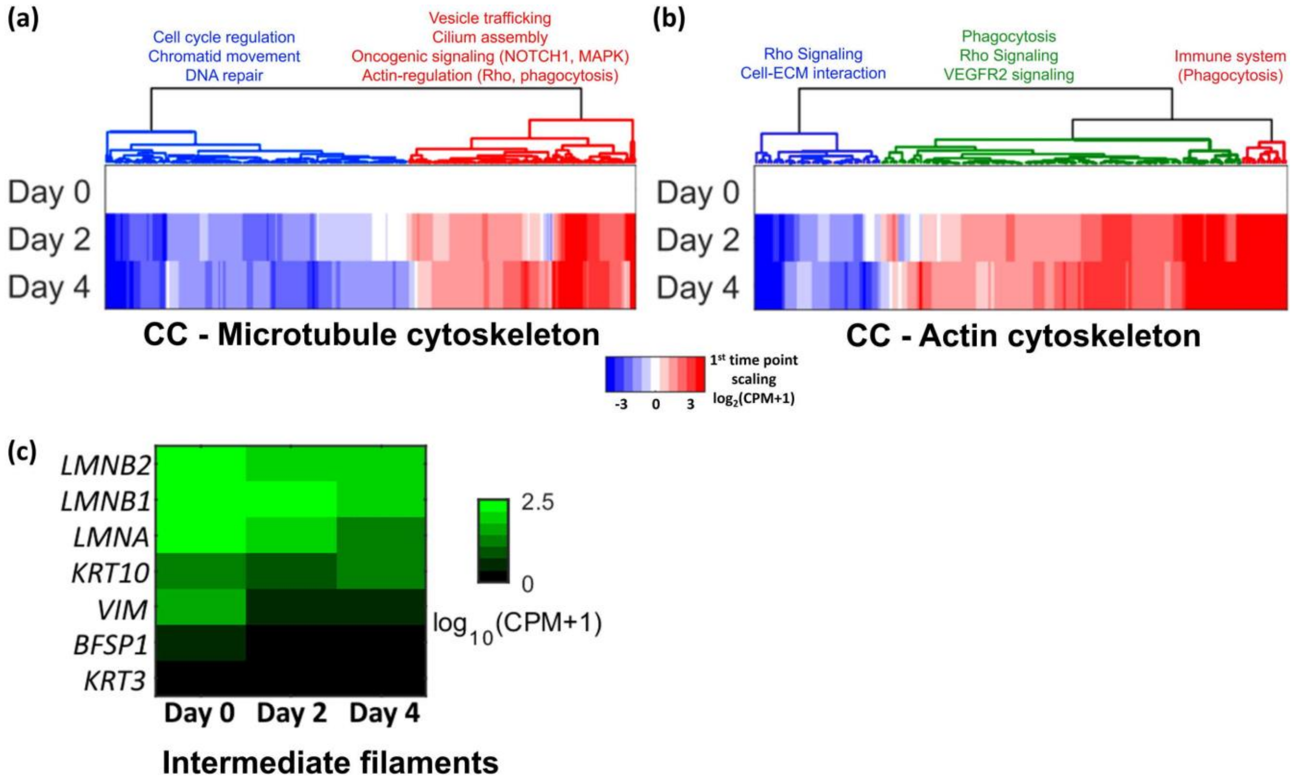

Figure S8 (a-b) Heat-map of DEGs within the reference Cellular Component category. Tree-clustering of genes according to expression pattern and main Reactome categories for color-coded gene groups. (c) Transcript levels of all intermediate filament genes detected in RNAseq data.

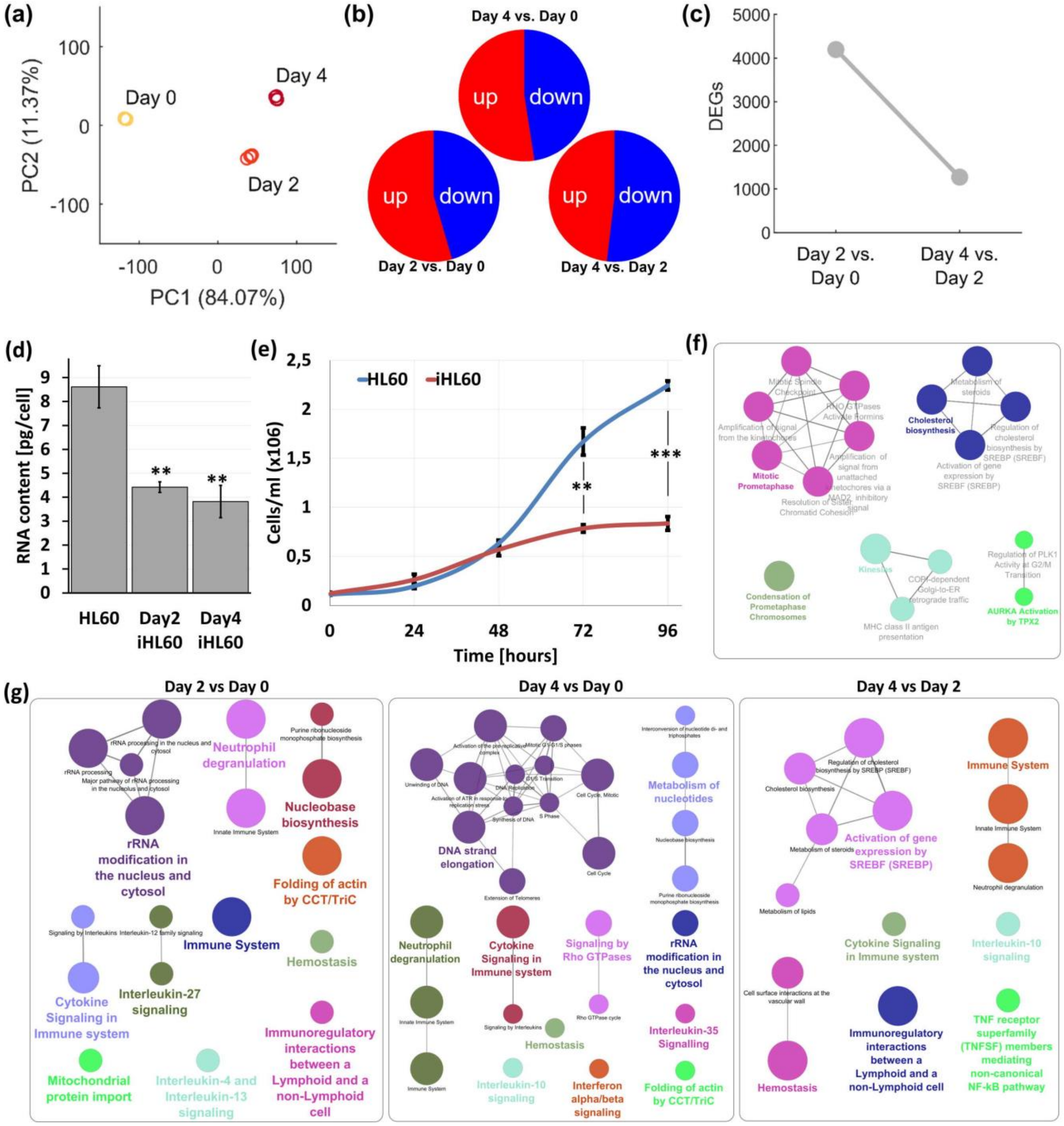

Figure S9 **(a)** PCA showing sample separation and reproducibility. **(b-c)** Summary of differentially expressed genes (DEGs) in various comparisons. **(d)** Total RNA quantification *per* cell at three time-points of iHL60 differentiation (n = 4). **(e)** Proliferation curve of HL60 and iHL60 (n = 3 independent cultures). **(f)** Annotated Figure 2F. **(g)** Reactome categories of up- and down-regulated DEGs at the specified comparisons.

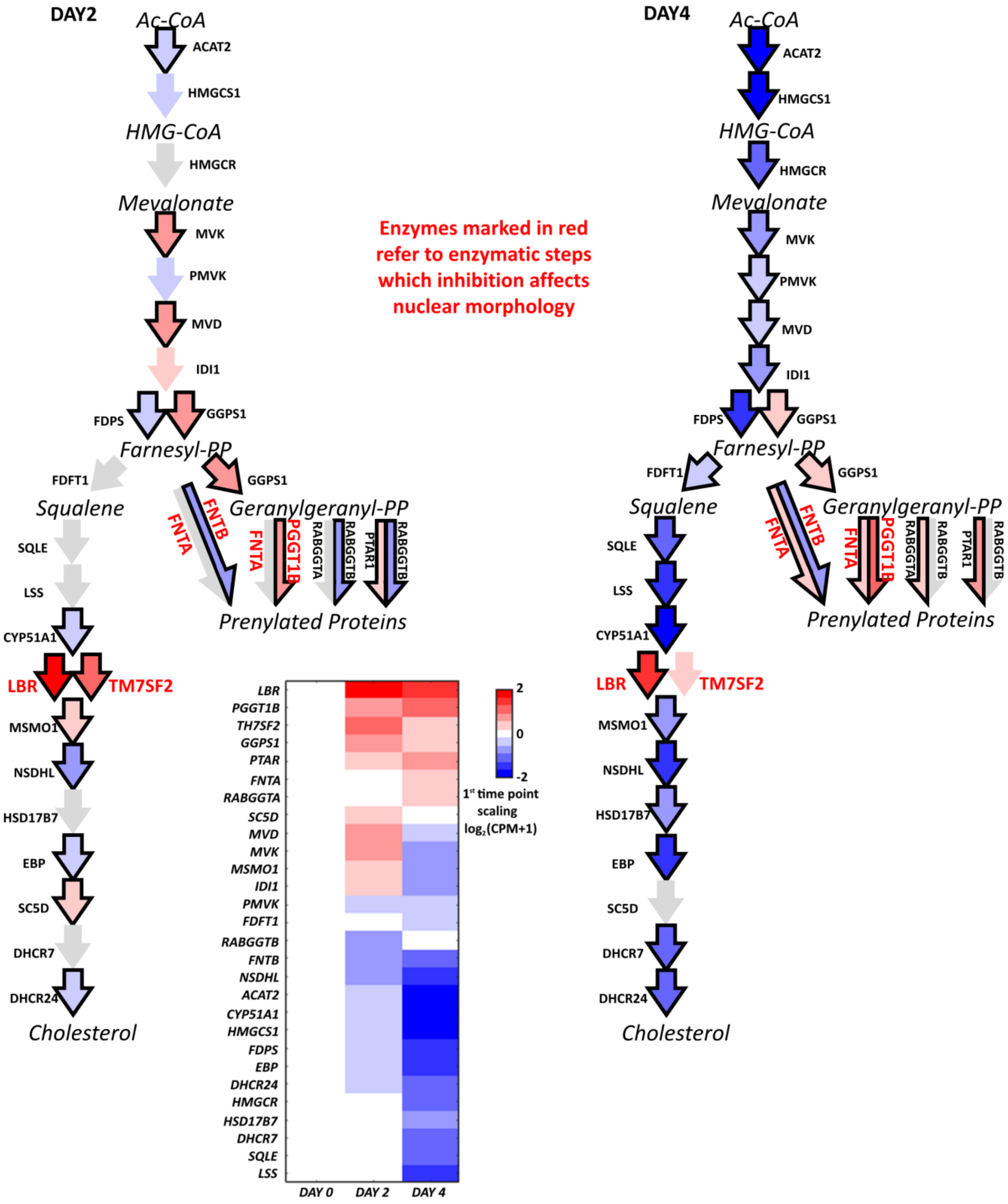

Figure S10 Annotated Figure 4 A with gene symbols and expression heatmap.

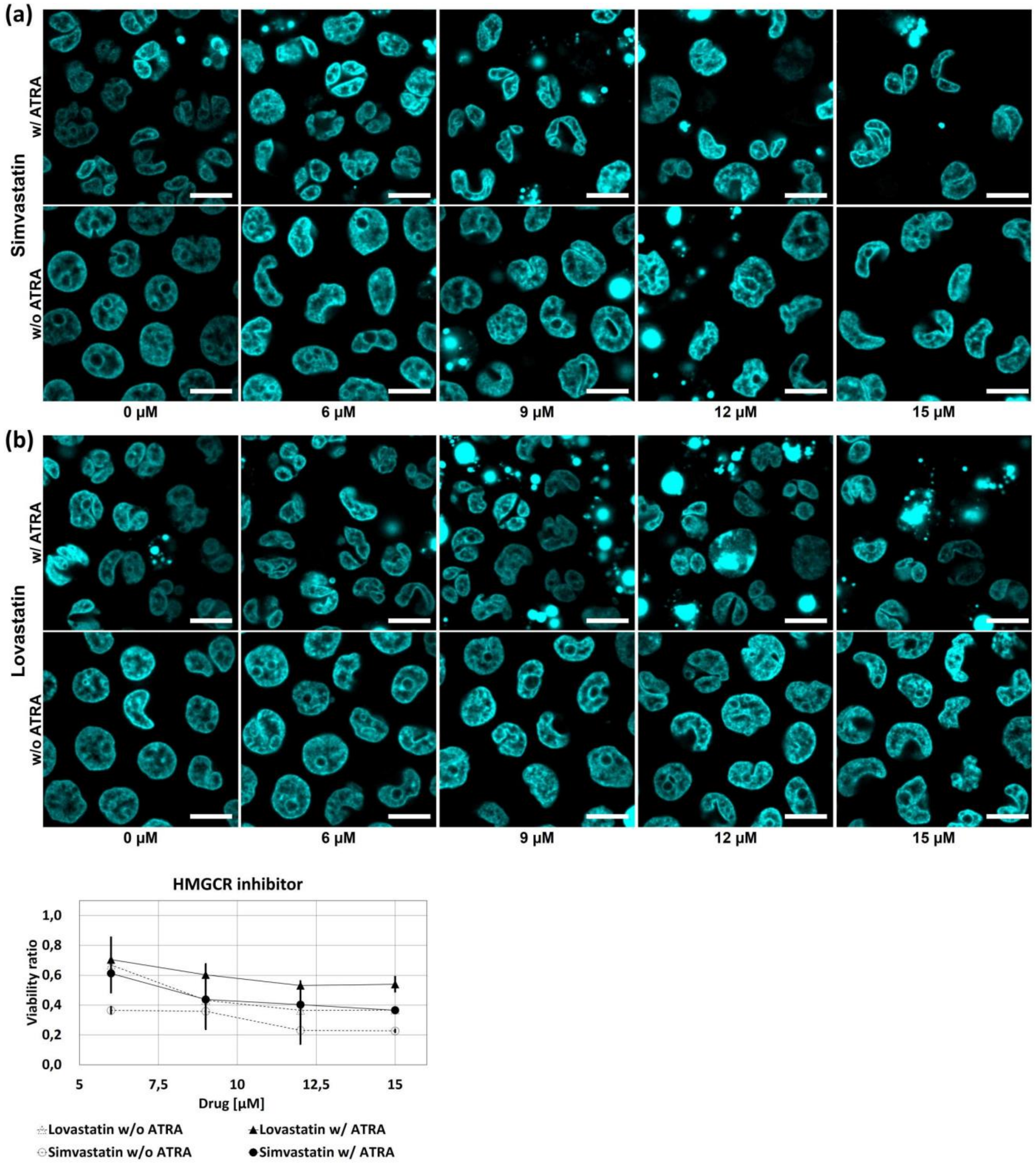

Figure S11 Live DNA staining of Day 3 iHL60 (scale bar = 10 µm), differentiated in presence of the indicated drugs at various concentrations and quantification of cell proliferation upon treatment expressed as ratio to final cell count in untreated controls. Viability data on n = 2 independent differentiation protocols.

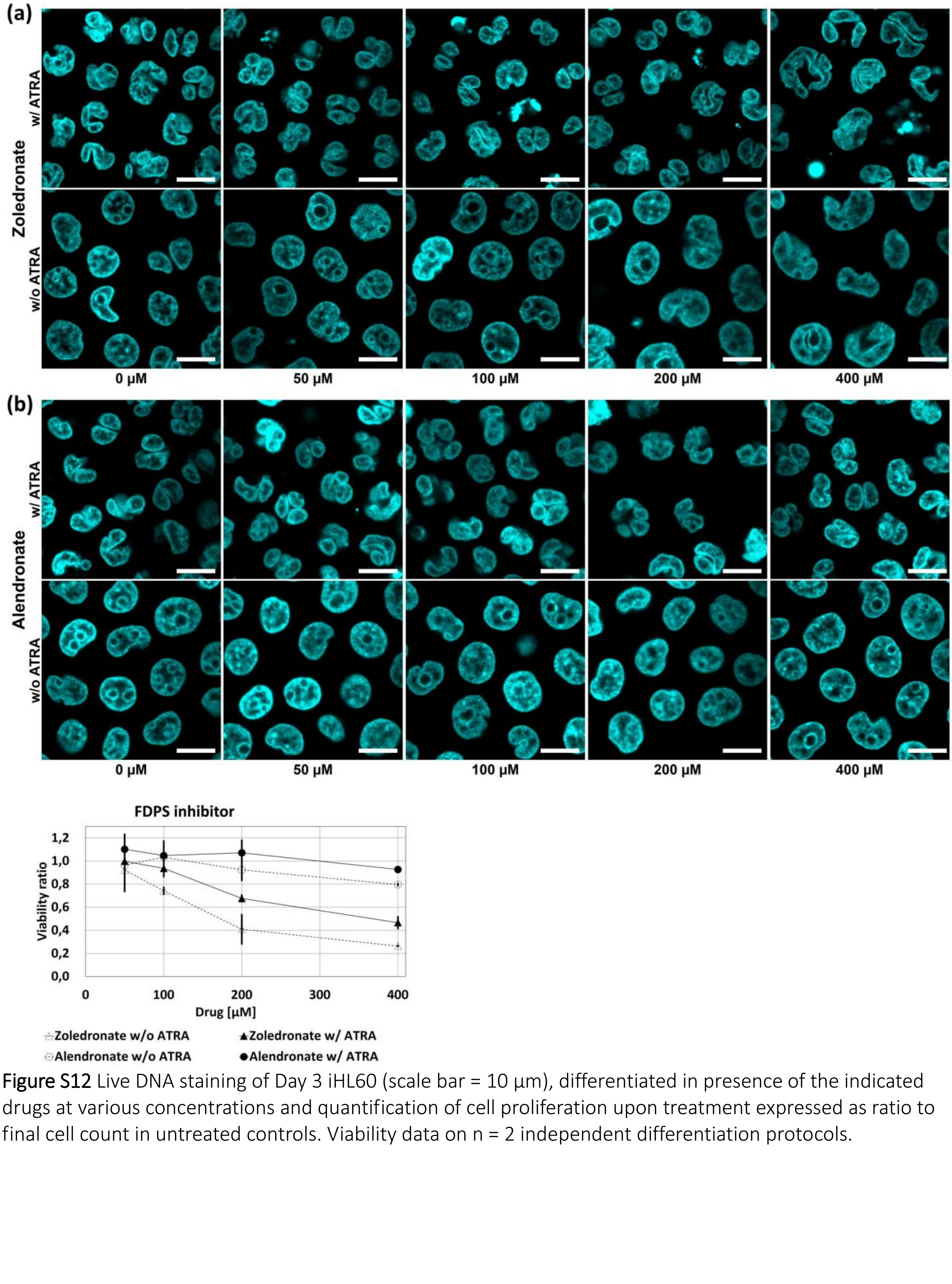

Figure S12 Live DNA staining of Day 3 iHL60 (scale bar = 10 µm), differentiated in presence of the indicated drugs at various concentrations and quantification of cell proliferation upon treatment expressed as ratio to final cell count in untreated controls. Viability data on n = 2 independent differentiation protocols.

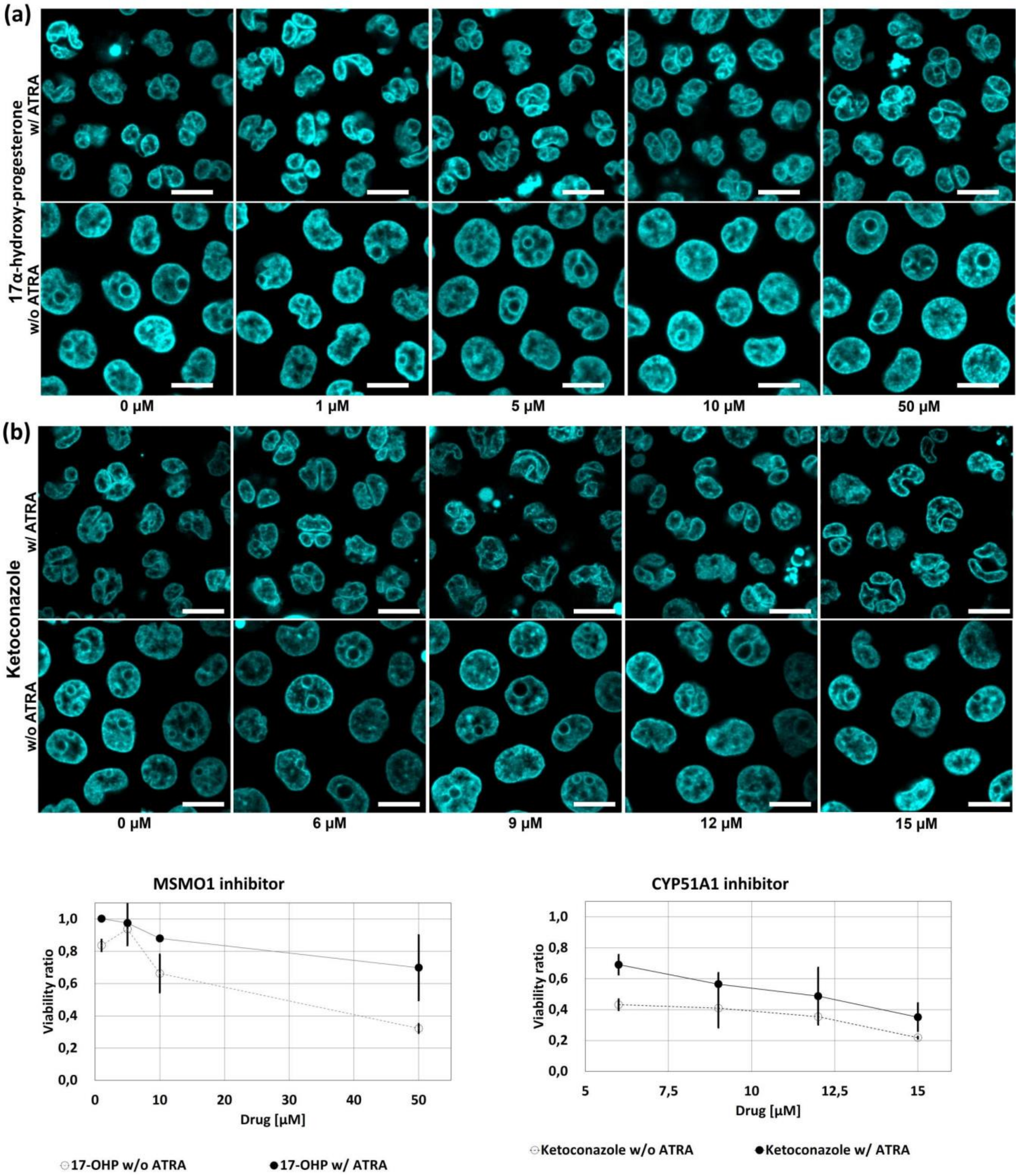

Figure S13 Live DNA staining of Day 3 iHL60 (scale bar = 10 µm), differentiated in presence of the indicated drugs at various concentrations and quantification of cell proliferation upon treatment expressed as ratio to final cell count in untreated controls. Viability data on n = 2 independent differentiation protocols.

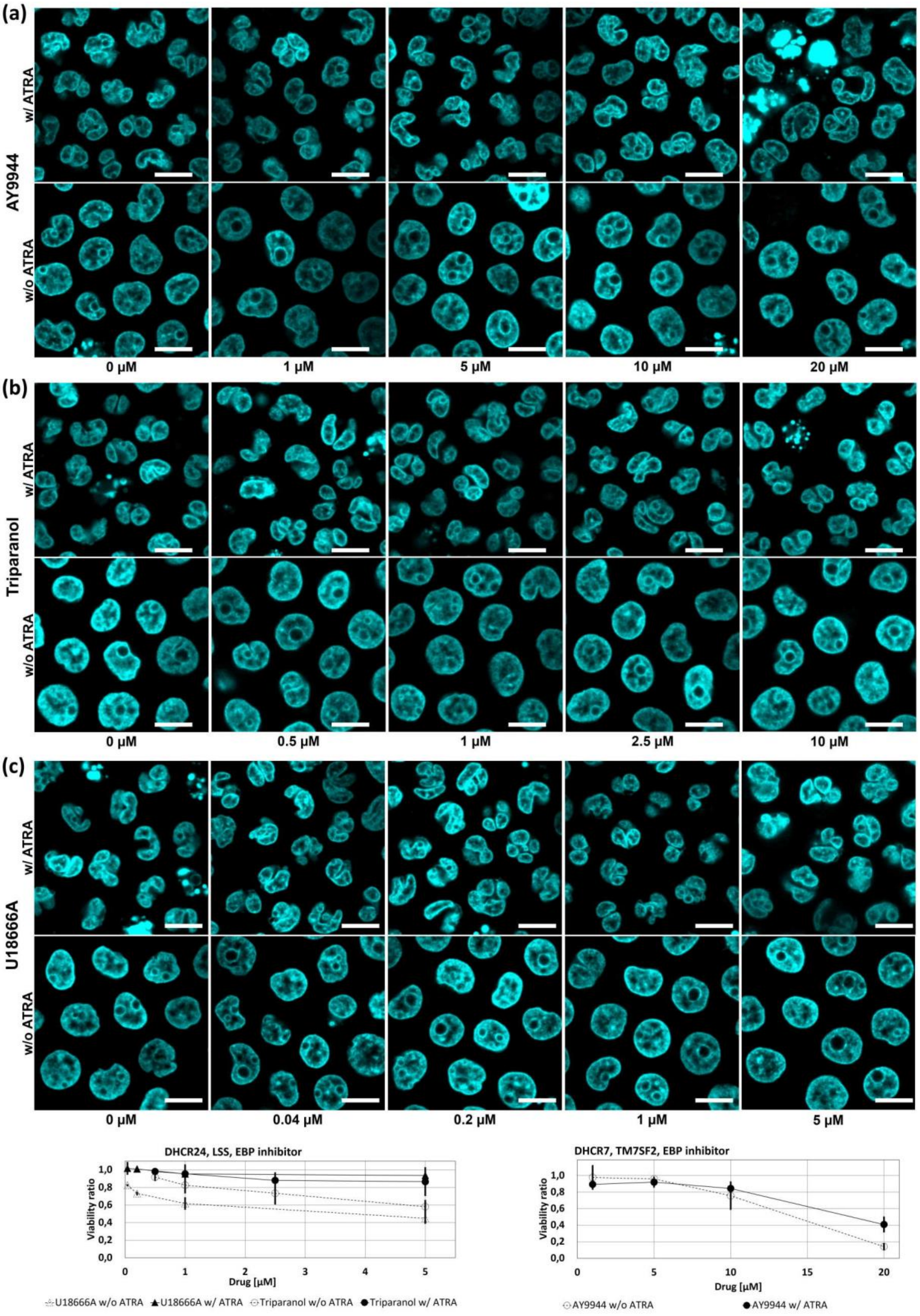

Figure S14 Live DNA staining of Day 3 iHL60 (scale bar = 10 µm), differentiated in presence of the indicated drugs at various concentrations and quantification of cell proliferation upon treatment expressed as ratio to final cell count in untreated controls. Viability data on n = 2 independent differentiation protocols.

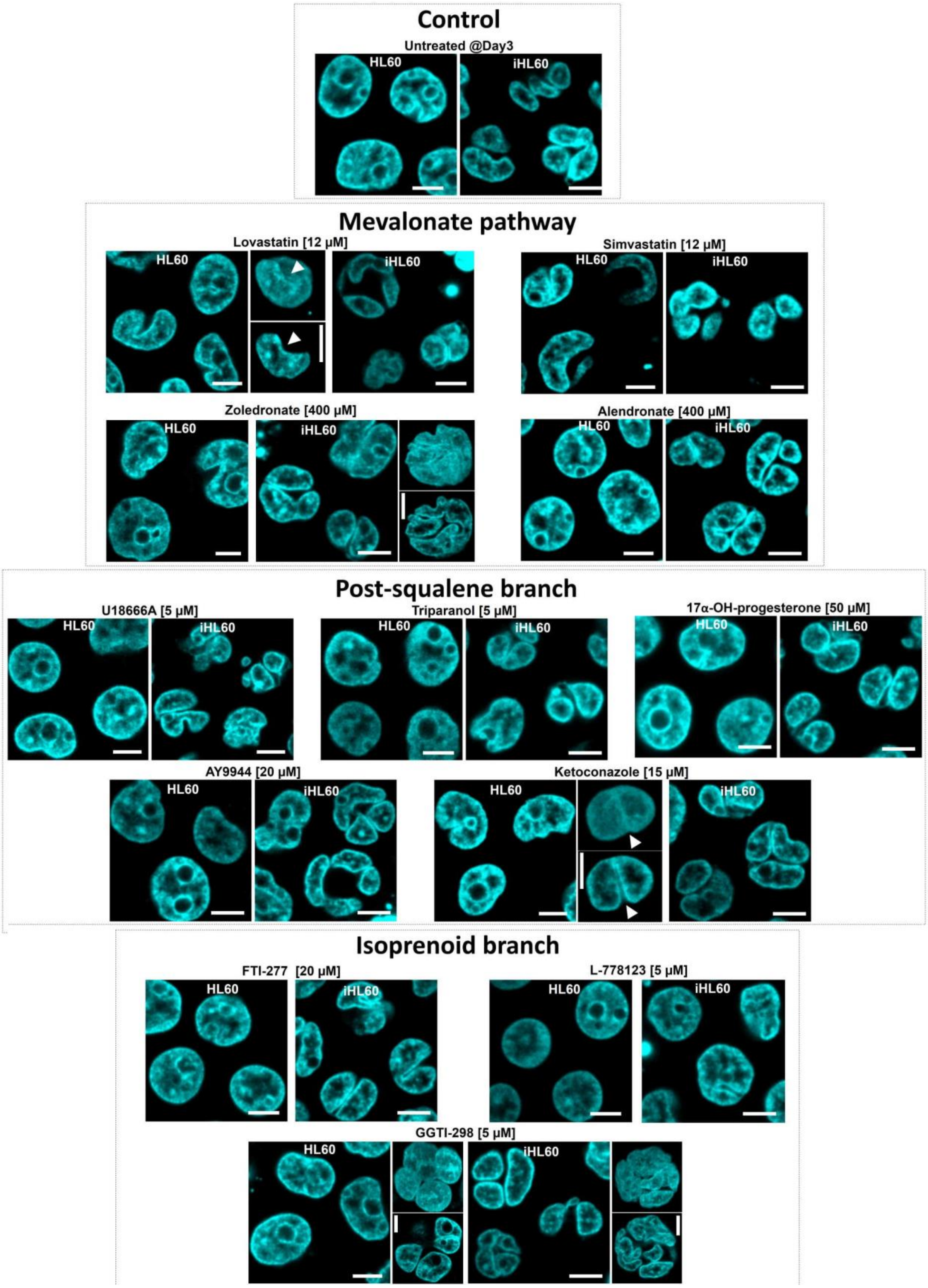

Figure S15 Live DNA staining of Day 3 iHL60 (scale bar = 10 µm, inset scale bar = 5 µm), differentiated in presence of the indicated drugs targeting **(a)** mevalonate branch, **(b)** post-squalene branch or **(c)** isoprenoid branch of the cholesterol biosynthetic pathway. Insets represent are 3D reconstructions (top) and central section (bottom).

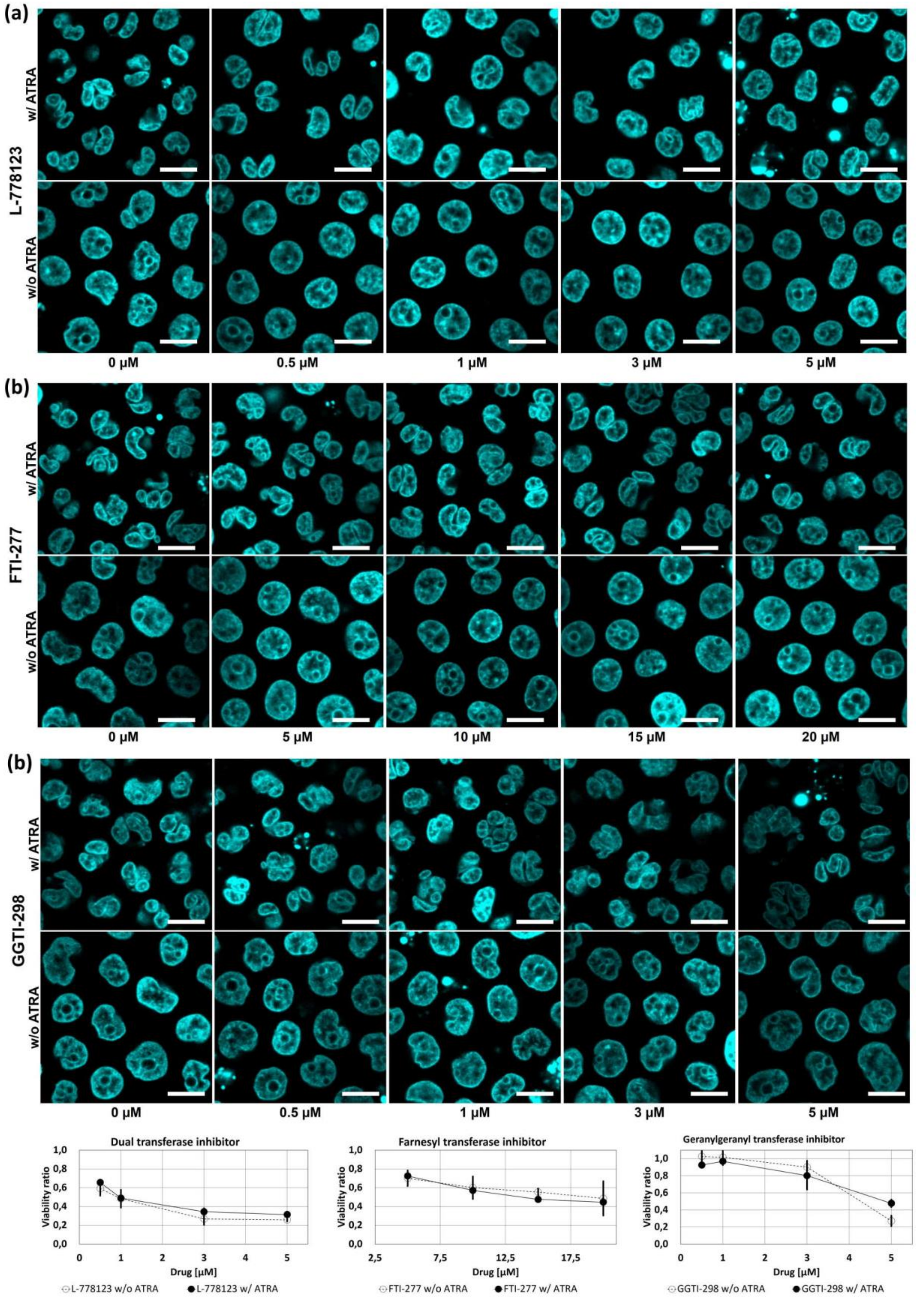

Figure S16 Live DNA staining of Day 3 iHL60 (scale bar = 10 µm), differentiated in presence of the indicated drugs at various concentrations and quantification of cell proliferation upon treatment expressed as ratio to final cell count in untreated controls. Viability data on n = 2 independent differentiation protocols.

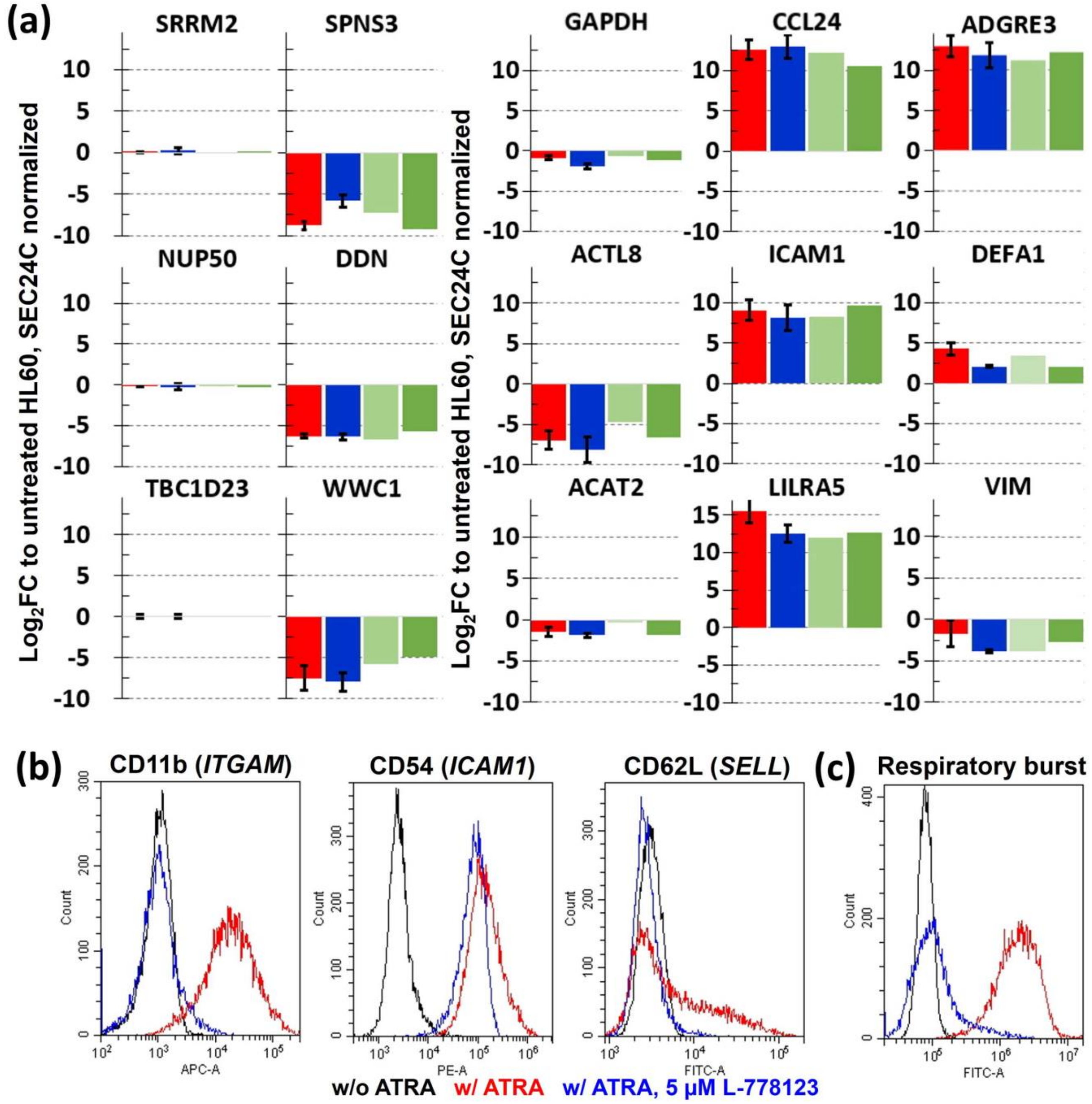

Figure S17 (a) mRNA expression of up-, down- and not-regulated genes in iHL60 at Day 3 (red = Untreated; blue = treated for 3 days with 5 uM L-778,123; light green = RNAseq data Day 2; dark green = RNAseq data Day 4. **(b)** Cytofluorimetric analyses for granulocyte-specific surface markers in iHL60 at Day 3 (color-coding maintained from description). **(c)** Cytofluorimetric assay for respiratory burst in iHL60 at Day 3 after PMA treatment (color-coding maintained from description).

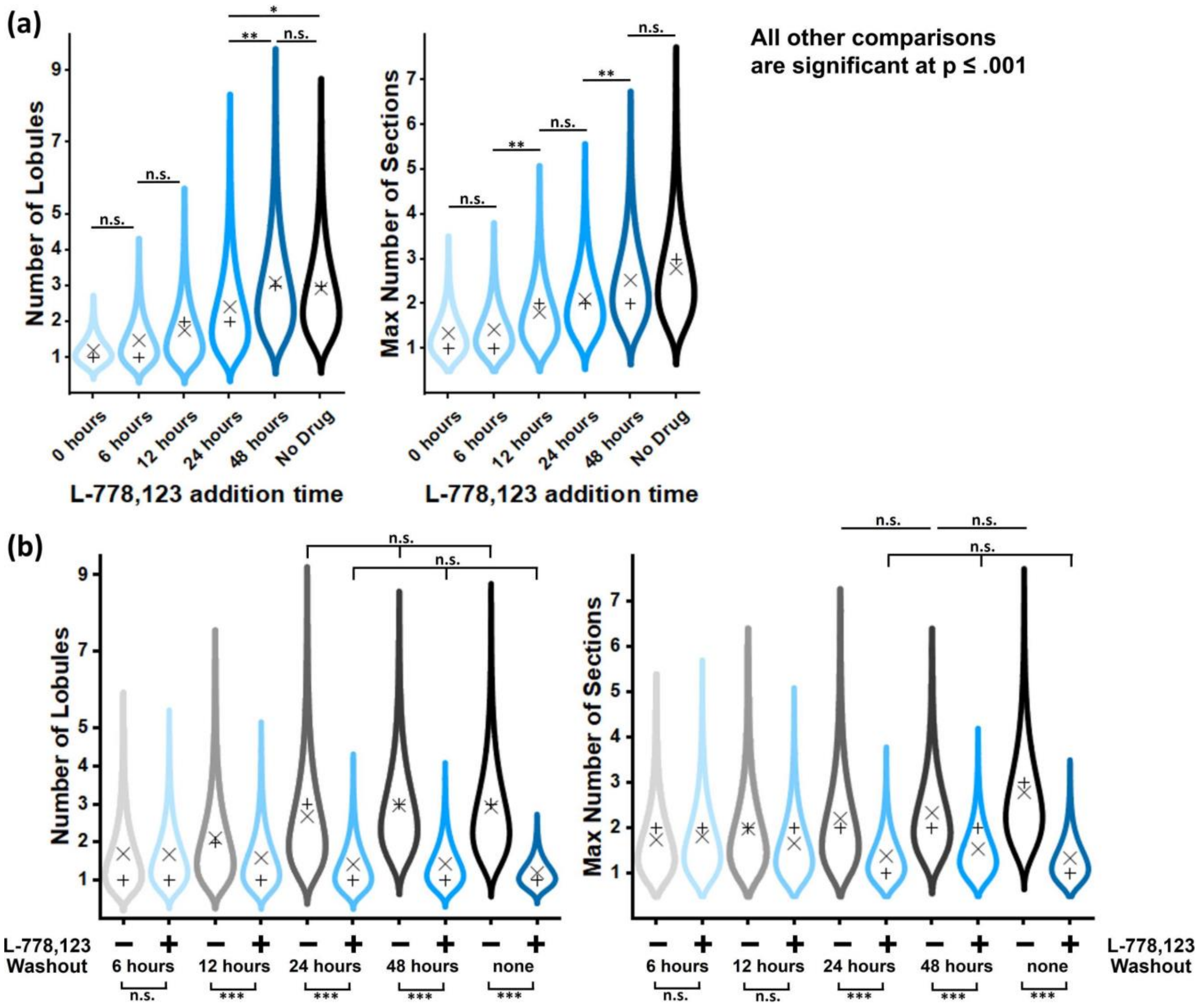

Figure S18 (a) Quantification of number of lobules per nucleus (minimum of cells quantified per condition = 128) and maximal number of sections per nucleus (minimum of cells quantified per condition = 102) in Day 3 iHL60, untreated and treated with L-778,123 added at indicated time-points (× = mean; + = median; n.s. = not significant, * = p<.05, ** = p<.01, Kruskal-Wallis test with Dunn post-hoc comparison). (b) Quantification of number of lobules per nucleus (minimum of cells quantified per condition = 127) and maximal number of sections per nucleus (minimum of cells quantified per condition = 135) in Day 3 iHL60, untreated and treated with L-778,123 followed by washout at the indicated time-point (× = mean; + = median; n.s. = not significant, *** = p<.001, Kruskal-Wallis test with Dunn post-hoc comparison).

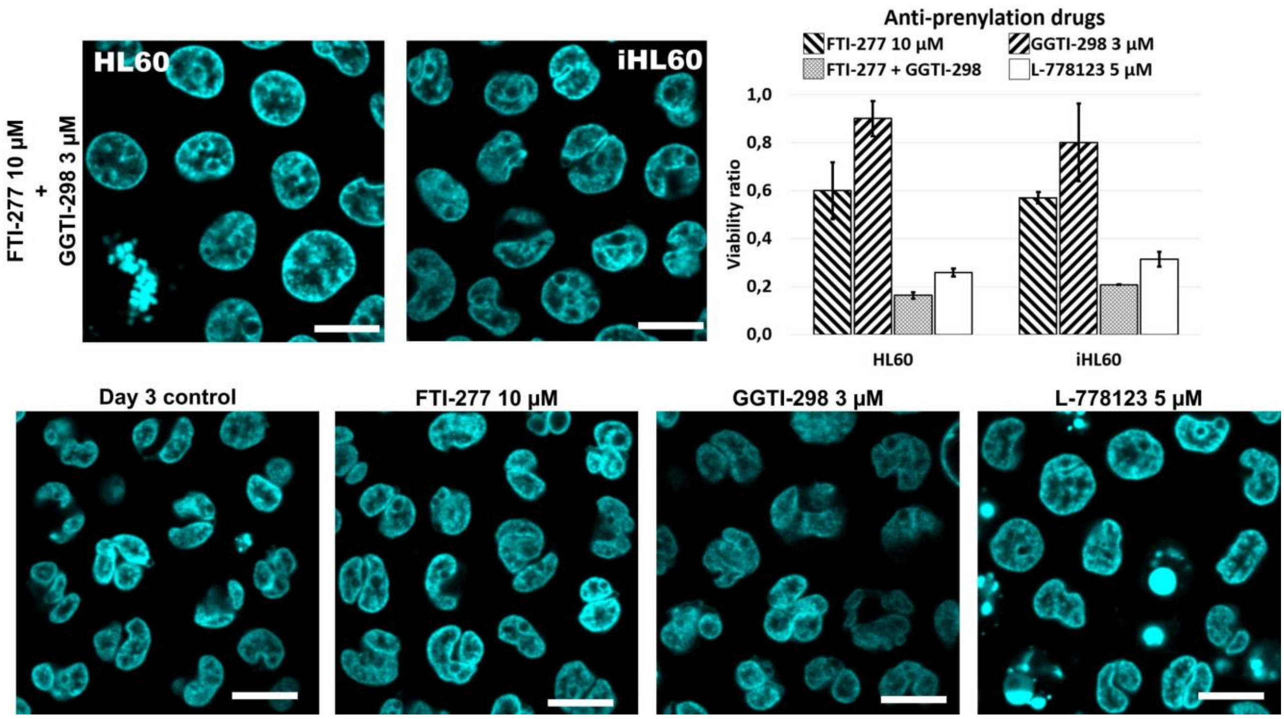

Figure S19 Live DNA staining of Day 3 iHL60 (scale bar = 10 µm), differentiated in presence of the indicated drugs and quantification of cell proliferation upon treatment expressed as ration to untreated controls (n = 3).

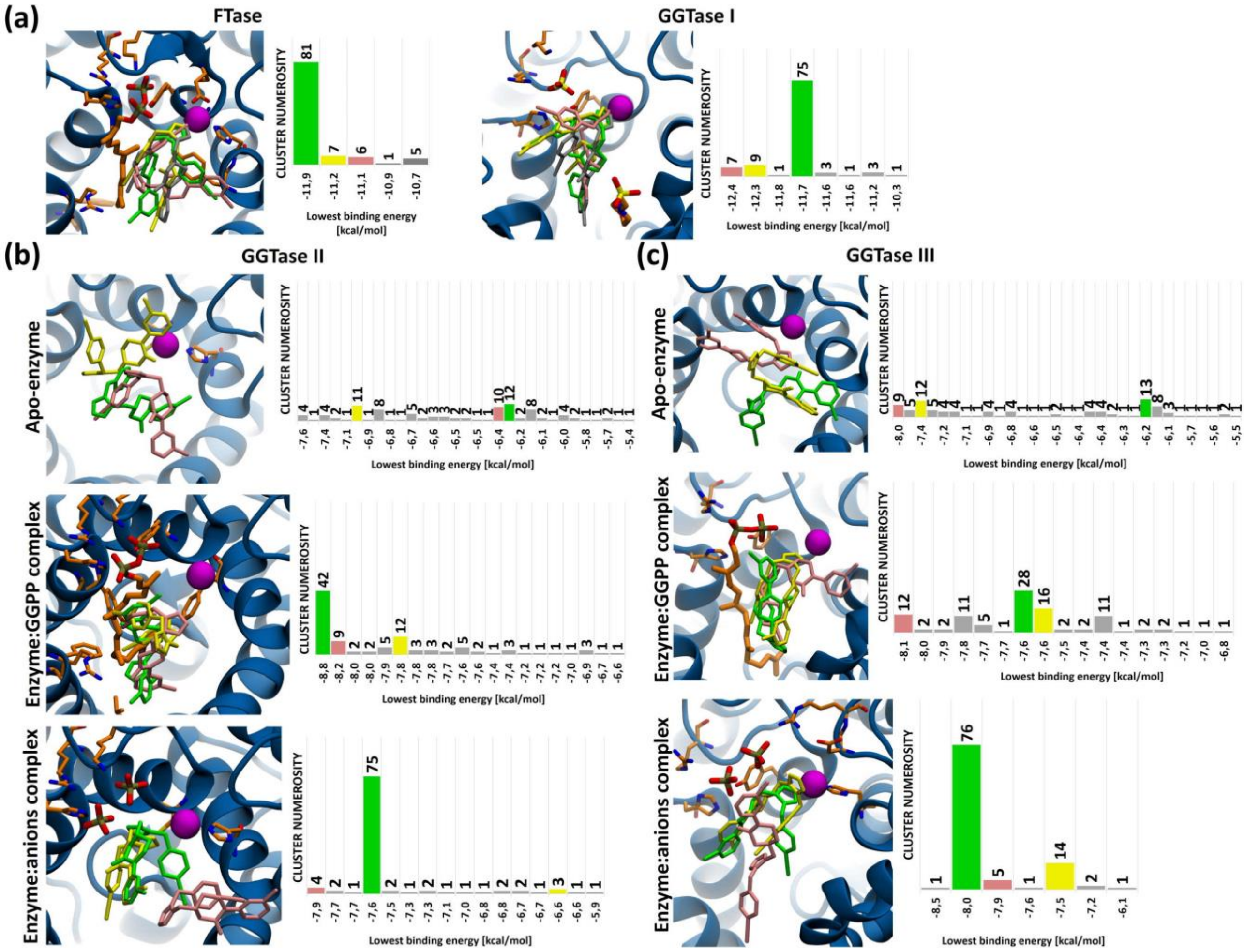

Figure S20 Molecular docking of L-778,123 to the references prenyl-transferases. The three most common poses are reported with conserved color-coding relative to cluster numerosity data. In **(a)** crystallized pose for L-778,123 is shown in grey.

Target	Forward sequence	Reverse sequence
ACTL8	CGACATTTGCCATCCTGACACC	CTCCCGTCTGTGGTTCTCCAA
ACAT2	GTTAGACAAGCCAGTGTGGGTG	CCTGTTCTCAAGTAAGCCAAGTGA
ADGRE3	TTCACCATCATCAACAGCCTCC	CCTGGAAAAACATCCCCCTCAC
CCL24	AACCAAACCACCTGCTAATCCC	AACCACATCACCTGCTCCCTC
DDN	CCAGCAACAACCAACAAAGCAG	TGTCAGAGACGGAAGGATGGAC
DEFA1	TCCCAGAAGTGGTTGTTTCCCT	AGCAGCAGAATGCCCAGAGT
GAPDH	GCTCTCTGCTCCTCCTGTTC	ACCAAATCCGTTGACTCCGA
ICAM1	CCGTGAATGTGCTCTCCCCC	CGTGGCTTGTGTGTTCGGTTTC
ITGAM	TGCTTACTTGGGTTATGCTGCC	GCCCTTGACATTAGCGTTGGAC
ITGB2	GGATGAGAGCCGAGAGTGTGT	TCAGAGCCTTCCAGATGACCAG
JAML	CACTGAAACTCATCCTGCTGCC	TATTCGTCCTTGGCGTGCTCT
LBR	ACTTGGTGATGCTTTTGGCTGA	GTCGTCAACAACGCTTCCTCAT
LILRA5	CTGGGACTGCCTCACACCTTC	TTGTTCTCTCTTCTGTTCACCTTCC
LMNA	CCTCATCCCTAAACAGCAAGCC	TACATCAAGTCTCCCAGTCGGT
LMNB1	AGAAGGCGAAGAAGAGAGGTTGA	TGAGGCGGAATGAGAGATGCTAA
LMNB2	AGTTTTCCCGCAGTTCAGTTCC	CCTCATTCTTCCTTCCCCAGGT
NUP50	GGAAACAACATAACCAGTGCCCC	GCAGCAAGAGAACCAAAGGCTAC
SEC24C	CTTCCTTTCAGGTGGAGAACGAC	GACCCGCATCACAGCATCAA
SPNS3	TCTTGGCTCTTCTTCCTGTCCC	CAGTTCCCAGTCAGCATCGTCA
SRRM2	AGAAGAATGAAAGACTCCGTGCTG	CTCCCGAACAAGGCTGTAAGGT
TBC1D23	TGGGGATGAAGAAGAATACGACACA	GTAACCAACAGATGACTGGGAAACA
VIM	GAAATGGCTCGTCACCTTCGT	AGAAATCCTGCTCTCCTCGCC
WWC1	CCACTCTGTCCAAAAAGCCACC	GCAGGTCTAACTCCAGGTCCA

Table S1 Primer sequences employed for real time PCR procedures.

Target	Molecule	Symbol	Supplier	References
Actin microfilaments	Latrunculin A	LatA	Sigma-Aldrich	(Chu et al., 2017; Maninova et al., 2017)
Actin microfilaments	Cytochalasin D	CytD	Cayman	(Olins et al., 2000; Olins and Olins, 2004)
Actin microfilaments	Jasplakinolide	Jasp	Cayman	(Makhija et al., 2016; Neubert et al., 2018)
Microtubules	Nocodazole	Noco	Sigma-Aldrich	(Olins et al., 2000; Olins and Olins, 2004)
Microtubules	Vincristine sulfate	VNCT	Selleck	(Barreca et al., 2020)
Microtubules	Paclitaxel	Pax	Selleck	(Kaya and Donmez, 2020; Ramdas et al., 2015)
Vimentin filaments	Withaferin A	WTA	Sigma-Aldrich	(Mohan and Bargagna-Mohan, 2016)
Intermediate filaments	Acrylamide	AA	Sigma-Aldrich	(Resutek et al., 2019; Sickles et al., 2007; Chen et al., 2016)
Intermediate filaments	β-β'iminodipropionitrile	IDPN	Sigma-Aldrich	(Feuilloley et al., 1988; Galigniana et al., 1998)
RhoA kinase (ROCK)	Y-27632	Y27632	Selleck	(Breuzart et al., 2019; Neubert et al., 2018)
Myosin I	Pentacholoropseudilin	PCIP	Aobious	(Bond et al., 2013)
Myosin II	Blebbistatin	Blebb	Selleck	(Bond et al., 2013)
Myosin V	Pentabromopseudilin	PBP	Sigma-Aldrich	(Bond et al., 2013)
Myosin VI	2,4,6-Triiodophenol	TIP	Bidepharm	(Bond et al., 2013)

Gene symbol	Enzymatic activity	Other synonyms	Inhibitor	Supplier	References
HMGCR	3-hydroxy-3-methyl-glutaryl-coenzyme A reductase	HMG-CoA reductase	Lovastatin, Simvastatin	Selleck, Selleck	(Newman et al., 1997)
FDPS	Dimethylallyltrans transferase	Farnesyl diphosphate synthase, FPPS, DMATT	Alendronate, Zoledronate	Selleck, Selleck	(Bergstrom et al., 2000; Kuroda et al., 2003)
LSS	2,3-oxidosqualene cyclase	Lanosterol synthase	Triparanol, U18666A	Selleck, Selleck	(Cenedella, 2009; Müller et al., 2017)
CYP51A1	lanosterol 14α-methyl demethylase	cytochrome P450 family 51 subfamily A polypeptide 1, 14α-DM	Ketoconazole	Selleck	(Sukhanova et al., 2013)
TM7SF2	sterol Δ14-reductase	DHCR14; transmembrane 7 superfamily member 2,	AY9944	Selleck	(Fernandez et al., 2005; Gatticchi et al., 2017)
MSMO1	sterol-4α-methyloxidase	C4-demethylase; SC4MOL	17α-Hydroxy-progesterone (17-OHP)	APExBIO	(Gatticchi et al., 2017; Lindenthal et al., 2001)
EBP	3β-hydroxysteroid-Δ8-Δ7 sterol isomerase	emopamil-binding protein; sigma receptor	Triparanol, U18666A, AY9944	Selleck, Selleck, Selleck	(Cenedella, 2009; Fernandez et al., 2005; Gatticchi et al., 2017; Müller et al., 2017)
DHCR7	sterol Δ7-reductase	7-Dehydrocholesterol reductase	AY9944	Selleck	(Fernandez et al., 2005; Gatticchi et al., 2017)
DHCR24	sterol Δ24-reductase	desmosterol reductase; 24-Dehydrocholesterol reductase; lanosterol 24-reductase	Triparanol, U18666A	Selleck, Selleck	(Cenedella, 2009; Müller et al., 2017)
FNTB	Farnesyl transferase	FTase	FTI-277, L-778123	Sellek, Targetmol	(Reid et al., 2004; Storck et al., 2019)
PGGT1B	Geranylgeranyl transferase	GGTase I	GGTI-298, L-778123	Sellek, Targetmol	(Reid et al., 2004; Tamanoi and Lu, 2013)

Table S2 Summary of all the small molecules employed in the study, with suppliers and references for each drug previously employed on HL60 cell system.
